# Toughening Mechanism in Nanotwinned Boron Carbide: A Molecular Dynamics Study

**DOI:** 10.3390/nano14181493

**Published:** 2024-09-14

**Authors:** Hongchi Zhang, Yesheng Zhong, Xiaoliang Ma, Lin Yang, Xiaodong He, Liping Shi

**Affiliations:** 1 Center for Composite Materials and Structures, Harbin Institute of Technology, Harbin 150080, China; zhanghongchi@hit.edu.cn (H.Z.); zhongyesheng@hit.edu.cn (Y.Z.); 22b918149@stu.hit.edu.cn (X.M.); linyang@hit.edu.cn (L.Y.); hexd@hit.edu.cn (X.H.); 2 Shenzhen STRONG Advanced Materials Research Institute Co., Ltd., Shenzhen 518000, China

**Keywords:** boron carbide ceramic, molecular dynamics, twin boundary, phase boundary, fracture toughness

## Abstract

Boron carbide ceramics are potentially ideal candidates for lightweight bulletproof armor, but their use is currently limited by their low fracture toughness. Recent experimental results have shown that sintered samples with high twin densities exhibit high fracture toughness, but the toughening mechanism and associated crack propagation process of nanotwinned boron carbide at the atomic scale remain a mystery. Reported here are molecular dynamics simulations with a reactive force field potential to investigate how nanoscale twins affect the fracture toughness of boron carbide ceramics. The results show that the strength disparity on either side of a twin boundary is the fundamental reason for the toughening effect; the twin boundary impedes crack propagation only when the crack moves to a region of higher fracture strength. The fracture toughness of nanotwinned boron carbide is greatly affected by the angle between the twin boundary and the prefabricated crack. At an angle of 120°, the twin boundary provides the maximum toughening effect, enhancing the toughness by 32.72%. Moreover, phase boundaries—another common structure in boron carbide ceramics—have no toughening effect. This study provides new insights into the design of boron carbide ceramics with high fracture toughness.

## 1. Introduction

Boron carbide (B_4_C) is one of the most important structural ceramic materials because of its outstanding physical and chemical properties, such as low density, superior hardness, high melting point, excellent chemical stability, and wear resistance [1,2,3,4]. In particular, its combination of low density and superior hardness makes B_4_C the best candidate among structural ceramics for lightweight ballistic armor [5,6]. Diffraction experiments [7] and electronic structure calculations [8] have indicated that the low density and superior hardness of B_4_C are due to its unique crystal structure and bonding properties. The first atomic model of B_4_C was proposed in 1959 by Silver and Bray [9], who reported a B_4_C unit cell in the form of a distorted cubic lattice composed of almost-regular icosahedrons and three-atom chains. However, deeper research has determined that the B_4_C unit cell is in fact a secondary structure composed of two primary structures: an icosahedron and a three-atom linear chain [10]. At the lattice level, B_4_C can be described as a rhombohedral or hexagonal unit cell, in which the three-atom chain is aligned along the [111] crystallographic direction in the rhombohedral unit cell and along the [0001] crystallographic direction in the hexagonal unit cell [11]. Given the fairly open space and strong covalent bonding inside its unit cell, B_4_C has a Hugoniot elastic limit (the yield point of uniaxial elastic compression) of up to 20 GPa; thus, it has an excellent hypervelocity impact resistance and a high pressure resistance [3,10,12,13]. However, experimental dynamics experiments have shown that B_4_C suffers from abnormal brittle fracture at impact speeds of 900 m/s or higher; this is characteristic of a low fracture toughness [14], and is incompatible with its high Hugoniot elastic limit [15,16]. In contrast, silicon carbide (SiC)—another commonly used lightweight armor material—does not suddenly lose its bulletproof properties when subjected to hypervelocity impact [17]. Therefore, improving the fracture toughness of B_4_C ceramics has become a prerequisite for their large-scale engineering application.

The previous mainstream view was that the fracture mode of B_4_C ceramics was predominantly transgranular fractures, as evidenced by the flat and smooth fracture surfaces observed microscopically [18]. However, findings from spark plasma sintering have shown that smaller grain sizes in fully dense B_4_C ceramics correlate with a higher fracture toughness [19,20]. Furthermore, images obtained using scanning electron microscopy reveal many rough areas smaller than 1 mm and the pullout of particles with a size of ca. 200 nm at fracture surfaces [21]. This indicates that intergranular fractures can also occur inside B_4_C, with crack deflection and bridging occurring around small grains. Therefore, the methods for toughening B_4_C ceramics can currently be divided into two types. The first method is the passivation of crack tips via the deflection, branching, and bridging of cracks, and the addition of second phases and grain boundary engineering are currently considered effective methods for crack-tip passivation in B_4_C ceramics [22,23,24]. Second-phase materials that have been used successfully include silicon [25]. non-oxide ceramics (e.g., SiC, TiB_2_, TiC) [26,27,28], graphene nanoplatelets [29,30], and reduced graphene oxide [31]. The second method is to enhance the inherent fracture toughness of B_4_C via microalloying, which uses foreign atoms to modify the three-atom chain structure connecting the icosahedron [32,33,34,35]. However, adding second phases breaks the combination of the low density and high hardness of B_4_C ceramics, and microalloying inevitably destroys the B_4_C crystal structure. Therefore, an alternative method must be found to improve the fracture toughness of B_4_C.

Research into superhard materials has discovered twin boundaries (TBs), which are unique structures with low interface energies that connect adjacent lattice regions in a mirror-symmetric manner [36]. The hardness and fracture toughness of diamond [37,38,39,40] and cubic boron nitride [41] with nanoscale twinned structures are much greater than those with the original structures. However, because of the complexity of the B_4_C crystal structure, its twinned structure was only recently discovered by using high-resolution scanning transmission electron microscopy [42]. The results from molecular dynamics (MD) simulations show that introducing TBs into B_4_C can narrow its amorphous shear bands and thereby increase its inherent hardness limit [43], and Ye and Wang [44] showed that a B_4_C sample containing high-density twins prepared via fast hot pressing sintering had improved fracture toughness. Furthermore, the stable phase of B_4_C contains many variations and various phase boundaries (PBs) due to the wide solid solubility of carbon (ca. 8–20 at.%) [10]. However, the toughening mechanism of TBs and the effect of PBs on fracture toughness remain unclear.

Although density functional theory is highly reliable, its simulation models are usually limited to a few hundred atoms and so cannot simulate the process of crack tip growth. By contrast, MD calculations based on empirical potentials can accurately simulate the mechanical behavior of large-scale models, so in the study reported herein, MD simulations were used to study the stress–strain relationships and fracture energy of twinned B_4_C during crack propagation. Next, how the angle between prefabricated cracks and TBs affected the fracture toughness of B_4_C was investigated. Finally, the influence of PBs on the fracture toughness of B_4_C was studied. This research provides a unique method for improving the fracture toughness of B_4_C ceramics to alleviate their abnormal brittle failure under hypervelocity impact.

## 2. Methodology

All the MD simulations reported herein were conducted using LAMMPS-2018 (Large-scale Atomic/Molecular Massively Parallel Simulator) [45] with the Reaxff (reactive forcefield) approach [46]. The Reaxff potential function has been used previously in accurate calculations of the dynamic behavior of B_4_C, such as its high-pressure deformation [47], nanoindentation [43], and high-speed impact [48]. The crack growth process of B_4_C was visualized using OVITO (Open Visualization Tool) [49].

**Figure 1:**
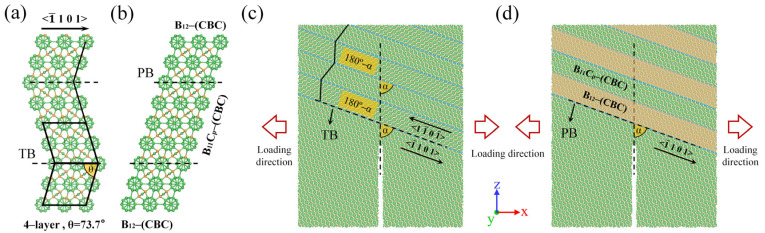
Left: atomic structures of (**a**) twin boundary (TB) and (**b**) phase boundary (PB). Right: simulation models for fracture toughness of (**c**) nanotwinned (NT) B_4_C and (**d**) PB B_4_C.

Figure 1a shows the atomic arrangement of the twin structure. As can be seen, the carbon atoms in the icosahedron still occupy polar sites after passing through the TB, resulting in both sides of the TB having the (B_11_Cp)-CBC crystal form. This arrangement of atoms in B_4_C is also called a symmetric twin [50]. B_4_C ceramics contain various PBs, but here, we discuss only two PB variants, i.e., (B_12_)-CBC and (B_11_Cp)-CBC, because these have the lowest energies among all possible variations of the basic B_4_C structure [51,52]. The (B_12_)-CBC variant is also called boron-rich B_4_C, the internal icosahedral structure of which is composed entirely of boron atoms. Figure 1b shows the atomic arrangement of the PB, the two sides of which are the (B_12_)-CBC phase and the (B_11_Cp)-CBC phase. For convenience, we refer to B_4_C containing TBs and PBs as nanotwinned (NT) B_4_C and PB B_4_C, respectively.

For the MD model of the equilibrium state, we first optimized the atomic positions and unit–cell parameters by using the conjugate gradient method [53]. Subsequently, an isothermal-isobaric (NPT) ensemble was used to relax the MD model at a temperature of 1 K using the Nosé–Hoover thermostat and barostat [54,55], thereby eliminating the effect of thermal fluctuations on atomic-scale fracture. For the simulations of crack propagation in B_4_C with different crack incident angles, the size of the MD model was 180 Å (X) × 10 Å (Y) × 200 Å (Z) and it comprised 58,000 atoms. The initial prefabricated crack length was 90 Å, and the included angle between the prefabricated crack and the TB or PB is defined as α, as shown in Figure 1c,d. As is well known, the force exerted on materials can cause cracks to propagate in three ways, i.e., opening (mode I), sliding (mode II), and tearing (mode III), with mode-I cracks being the most common and dangerous in engineering applications. Therefore, the propagation of a mode-I crack was simulated by applying a constant velocity to thin regions on the left and right sides of the MD model. To reduce the size effect, the MD models had periodic boundary conditions in the Y direction. All the MD simulations used a constant displacement rate of 5 × 10^−5^ Å/fs. It is worth noting that, when testing fracture toughness experimentally, the loading rate is usually slower than that of molecular dynamics. For example, Subhash et al. [56] used a loading rate of 0.1 μm/s when studying the fracture toughness of boron carbide-based ceramics. In order to investigate the effect of loading rate on the stress–strain relationship, different strain rates of 1 × 10^−5^ Å/fs, 2.5 × 10^−5^ Å/fs, and 5 × 10^−5^ Å/fs were imposed in the fracture model. The results show that the loading rate has little effect on the stress–strain relationship of boron carbide ceramics. Stress–strain curves for different loading rates are provided in the Supplementary Materials. To avoid the generation of stress waves, a linear velocity distribution was imposed on the free-zone atoms according to their positions.

An important indicator for measuring the fracture toughness of materials is the fracture energy, G, which represents the energy required for crack expansion per unit area, i.e.,

(1) $$G=H\int_{0}^{\varepsilon_{c}}\sigma d\varepsilon .$$

where H is the effective width of the MD model, and $\varepsilon_{c}$ is the strain at which the material breaks completely. Taking mode-I cracks as an example, the fracture energy (in units of J/m^2^) is the integral of the corresponding stress–strain curve multiplied by the effective width of the model [39]. Note that the calculated fracture energy includes not only the energy required to break the material to form a new surface but also the strain energy stored inside the material during the loading process; therefore, a more accurate definition is the work required to completely break the material.

## 3. Results and Discussion

### 3.1. Effect of TBs on Fracture Toughness

**Figure 2:**
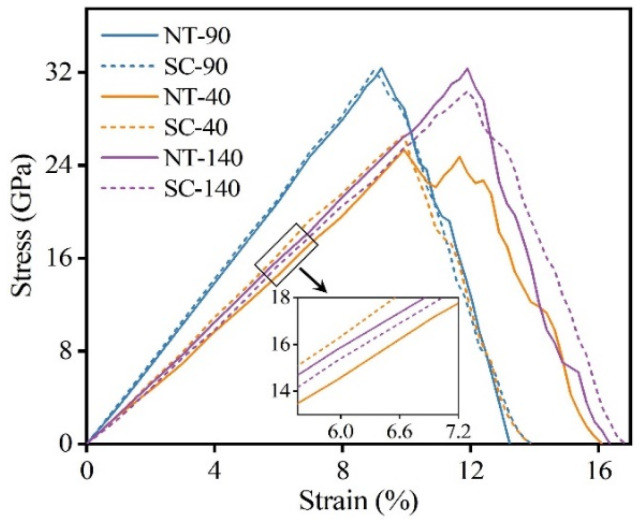
Stress–strain relationships of single-crystal (SC) and NT B_4_C at several typical included angles.

**Figure 3:**
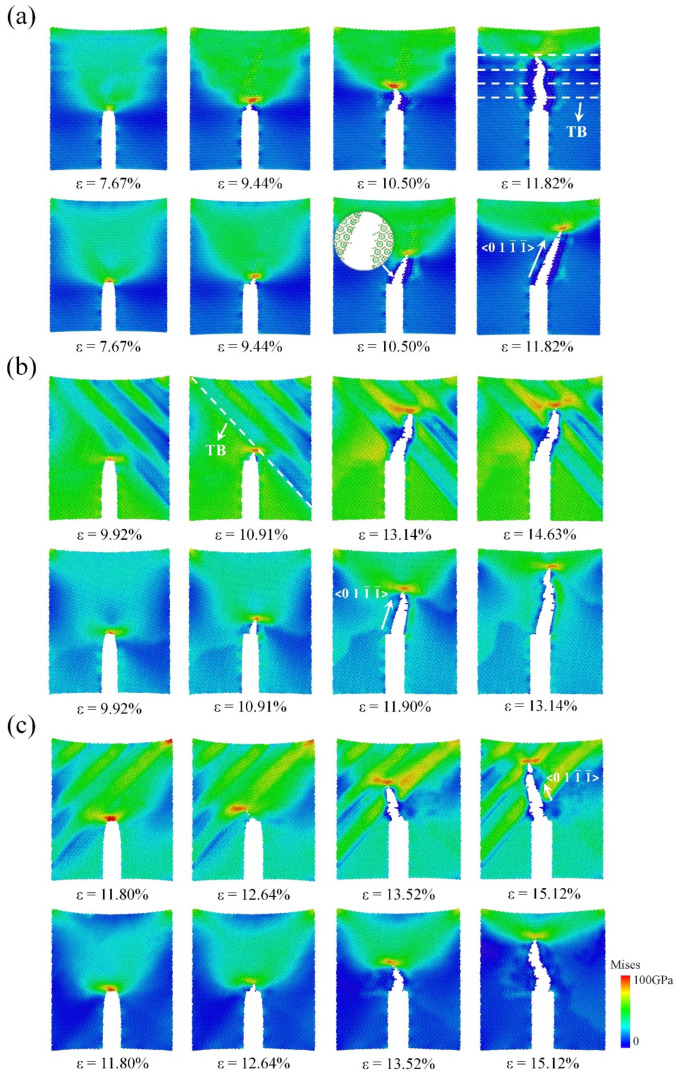
Changes in von Mises stress distribution with crack propagation for NT (first row) and SC (second row) B_4_C models at angles of (**a**) 90°, (**b**) 40°, and (**c**) 140°.

As shown in Figure 2, we obtained the stress–strain curves of single-crystal (SC) B_4_C and NT B_4_C at several typical included angles (40°, 90°, and 140°). The angle of 90° is the angle at which the prefabricated crack and the TB are perpendicular to each other. The angles of 40° and 140° are symmetrical about 90° and the two are complementary. In Figure 2, NT-90 indicates the model in which the included angle between the TB and the prefabricated crack is 90°, and SC-90 indicates the model in which the included angle between the <$\overset{-}{1}$101> crystal orientation of SC B_4_C and the prefabricated crack is 90°. The stress–strain curves indicate that the elastic modulus of NT-90 is nearly identical to that of SC-90; this is because (i) the material between adjacent TBs of NT B_4_C is still SC B_4_C and (ii) the SC B_4_C on both sides of a TB is complementary to the rotation angle, as shown in Figure 1b. Therefore, the fracture stress and fracture strain of NT-90 and SC-90 are also almost the same, suggesting that the presence of TBs has almost no effect on fracture toughness at this angle. Figure 3a shows the changes in the von Mises stress distribution of the NT-90 and SC-90 models with crack growth. The crack growth processes of the NT-90 and SC-90 models are basically the same because the angles of the SC B_4_C on both sides of the TB in the NT-90 model are consistent. Although the crack in the NT-90 model expands forward in a Z shape, the total length of the crack does not increase. Additionally, the crack propagation process is not hindered by the TB. Furthermore, the atomic configuration of the fracture surface shows that cracks tend to propagate from between the icosahedron and the three-atom chain structure rather than through the icosahedron. This is due to the difference in bonding strength within the icosahedron and between the icosahedron and the three-atom chain. Raman spectroscopy and DFT simulation results indicate that the bonding strength within the icosahedron is greater, which is also the fundamental reason for the high bulk modulus of B_4_C [57,58]. Therefore, cracks tend to propagate from the weaker bonding structures inside the unit cell, resulting in the crack propagating strictly along the <$01\overset{-}{1}\overset{-}{1}$> crystal orientation in both the NT-90 and SC-90 model.

Although the SC-40 model has a higher elastic modulus than that of the SC-140 model, it is less strong, indicating that the elastic modulus and strength of SC B_4_C show obvious anisotropy as the angle changes. The overall elastic modulus of the NT-40 model is slightly lower than that of the SC-40 model, resulting in lower stress thresholds at which prefabricated cracks begin to propagate as compared to SC B_4_C. The crack propagates first in SC-40, and when the crack propagates to the first TB, it encounters SC-140 with a higher fracture strength. Subsequently, the stress at the crack tip continues to accumulate until it reaches the fracture stress of SC-140, at which time the crack will continue to propagate forward. Hence, the length of crack propagation in the NT-40 model is shorter than that in the SC-40 model under the same strain, and the TB hinders the crack propagation to a certain extent, as shown in Figure 3b. It is worth noting that the Mises stress distributions in the NT-40 model and the SC-40 model shown in Figure 3b are significantly different. Firstly, the twin boundary of the NT-40 model is flanked by the SC-40 model and the SC-140 model. Since the elastic modulus of the SC-40 model is greater than that of the SC-140 model, the stress level of the SC-40 model is higher than that of the SC-140 model at the same fracture strain. Therefore, during the tensile fracture process, the NT-40 model shows alternating high and low stress regions on both sides of the twin boundary, namely blue-green stripes. Secondly, when the crack extends to the first twin boundary, the Mises stress at the crack tip of the NT-40 model is greater than that of the SC-40 model. This is because the SC-140 model on the other side of the twin boundary has a higher strength, so the crack tip continues to accumulate stress until it exceeds the strength of SC-140. Therefore, the number of highly stressed atoms near the twin boundary in the NT-40 model is higher than that in the SC-40 model. Note that not all TBs can hinder crack propagation; this occurs only when the crack enters from the low-strength SC B_4_C region to the high-strength SC B_4_C region, thereby forming an obstacle to the crack. Therefore, the stress–strain curve of the NT-40 model shows certain plastic characteristics, and when the stress reaches the maximum value, it changes to a wavy shape until the material breaks completely. For the tensile fracture at 140°, the fracture stress of the NT-140 model is slightly higher than that of the SC-140 model because the elastic modulus of the former is higher than that of the latter. However, the changing trends of the stress–strain curves of the two are almost the same, which shows that the presence of the TB has little effect on the fracture strain. This is because the crack propagation process of NT-140 goes from a region of high fracture strength to a region of low fracture strength, and this time the TB no longer hinders crack propagation.

**Figure 4:**
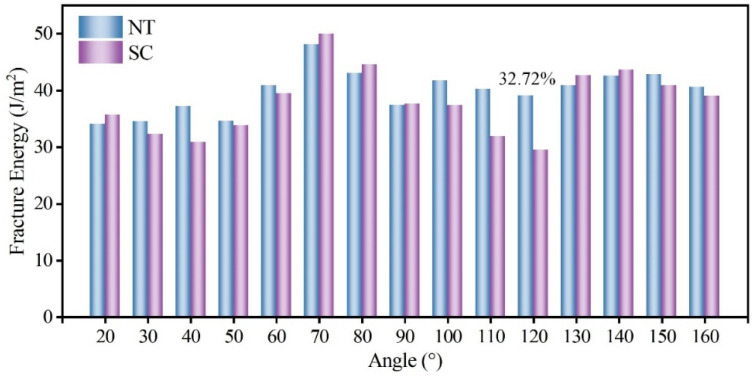
Variation in fracture energy of NT and SC B_4_C with included angle.

**Figure 5:**
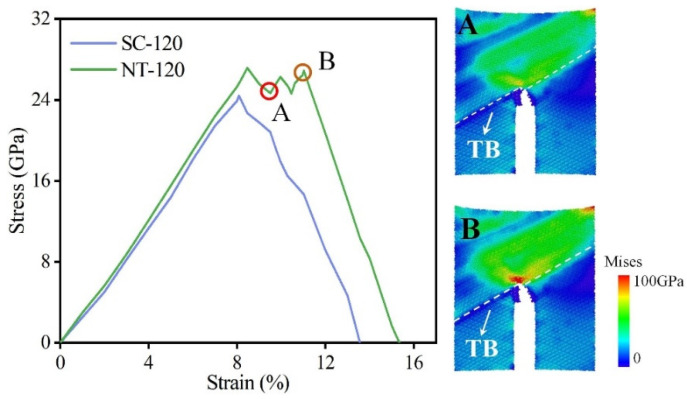
Stress–strain relationship of SC-120 model and NT-120 model.

Using the fracture energy introduced in Equation (1) as the indicator, the fracture toughness of NT B_4_C and SC B_4_C is calculated as a function of the included angle, as shown in Figure 4. The MD calculation results show that the fracture toughness of the two is greatly affected by the angle. The calculated maximum value of the fracture energy of SC B_4_C is 50.02 J/m^2^, the minimum value is 29.44 J/m^2^, and the maximum value is 1.70 times the minimum value. The fracture toughness of B_4_C measured in experiments is in the range of 2.9–3.7 MPa·m^0.5^ [59]. Its dispersion is also larger, which is consistent with the MD results. This is because the angles are random when the fracture toughness is measured experimentally, so the experimental measurements have a large dispersion. Furthermore, the maximum and minimum fracture energies of NT B_4_C are 48.10 J/m^2^ and 34.01 J/m^2^, respectively; the former is 1.41 times the latter, indicating that the uniformity of fracture toughness of NT B_4_C is better than that of SC B_4_C. The existing fracture toughness test results for NT B_4_C are in the range of 3.66–4.18 MPa·m^0.5^ [44,60], showing that introducing TBs improves the inherent fracture toughness of SC B_4_C. Our MD calculation results in Figure 4 also show that the presence of TBs has a toughening effect, which is consistent with the experimental results, except that the toughening effect is greatly affected by the included angle. At 40° or 100°–120°, the TB has a very obvious toughening effect, while at other angles there is no effect on the fracture toughness. When the included angle is 120°, the fracture toughness of single-crystal boron carbide is the smallest and the toughening effect of the twin boundary is the strongest, reaching 32.72%. The crack propagation direction of the SC-120 model is close to the prefabricated crack direction, so the SC-120 model has the shortest crack propagation path. Therefore, the SC-120 model has the lowest fracture toughness among all single-crystal models. We plotted the stress–strain curves of the SC-120 model and the NT-120 model during crack propagation, as shown in Figure 5. The elastic modulus of the NT-120 model is higher than that of the SC-120 model, resulting in a higher stress threshold for the start of prefabricated crack extension than that of the SC-120 model. When the stress at the crack tip in the NT-120 model reaches the strength of SC-120, the crack begins to propagate. Subsequently, when the fracture strain reaches 10.07% (point A in Figure 5), the crack propagates to the first twin boundary, at which point the crack propagation encounters an obstacle. As the fracture strain continues to increase, the stress at the crack tip continues to accumulate until it exceeds the strength of the SC-60 model on the other side of the twin boundary. The crack then continues to extend forward (point B in Figure 5), eventually leading to complete fracture of the model. From the Mises stress cloud diagrams at points A and B, it can be seen that the stress at the crack tip at point B is significantly higher than that at point A, which indicates that the twin boundary hinders the crack propagation. Fracture energy calculation shows that the toughening effect of the twin boundary is best when the angle is 120°. In addition, there is also an asymmetric twin form in B_4_C, which was first discovered by Fujita et al. [42]. Subsequently, Xie et al. [50] determined the precise atomic arrangement of asymmetric twins through DFT calculations. We calculated the effect of asymmetric twin on the fracture toughness of B_4_C at an angle of 120°. MD simulation results show that asymmetric twins have the same toughening effect on boron carbide and the toughening mechanism is the same as that of symmetric twins. The stress–strain curve for the asymmetric twin is provided in the Supplementary Materials.

### 3.2. Effect of PBs on Fracture Toughness

**Figure 6:**
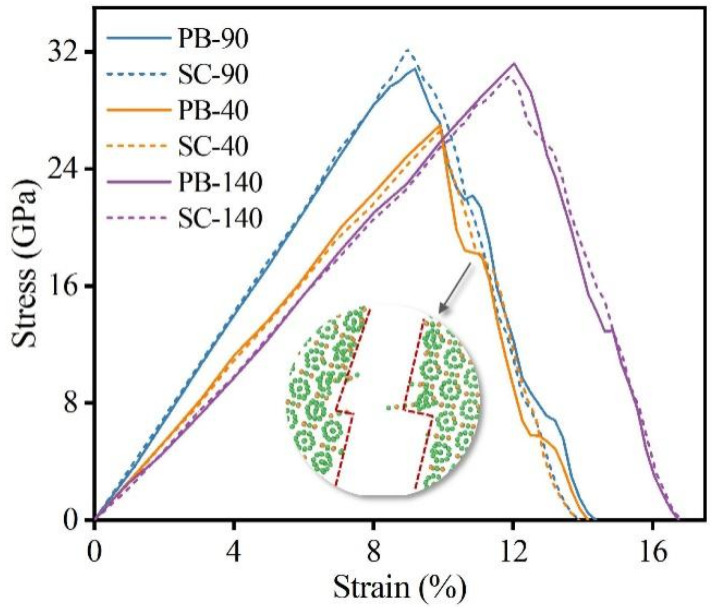
Stress–strain relationships of SC and PB B_4_C at several typical included angles.

**Figure 7:**
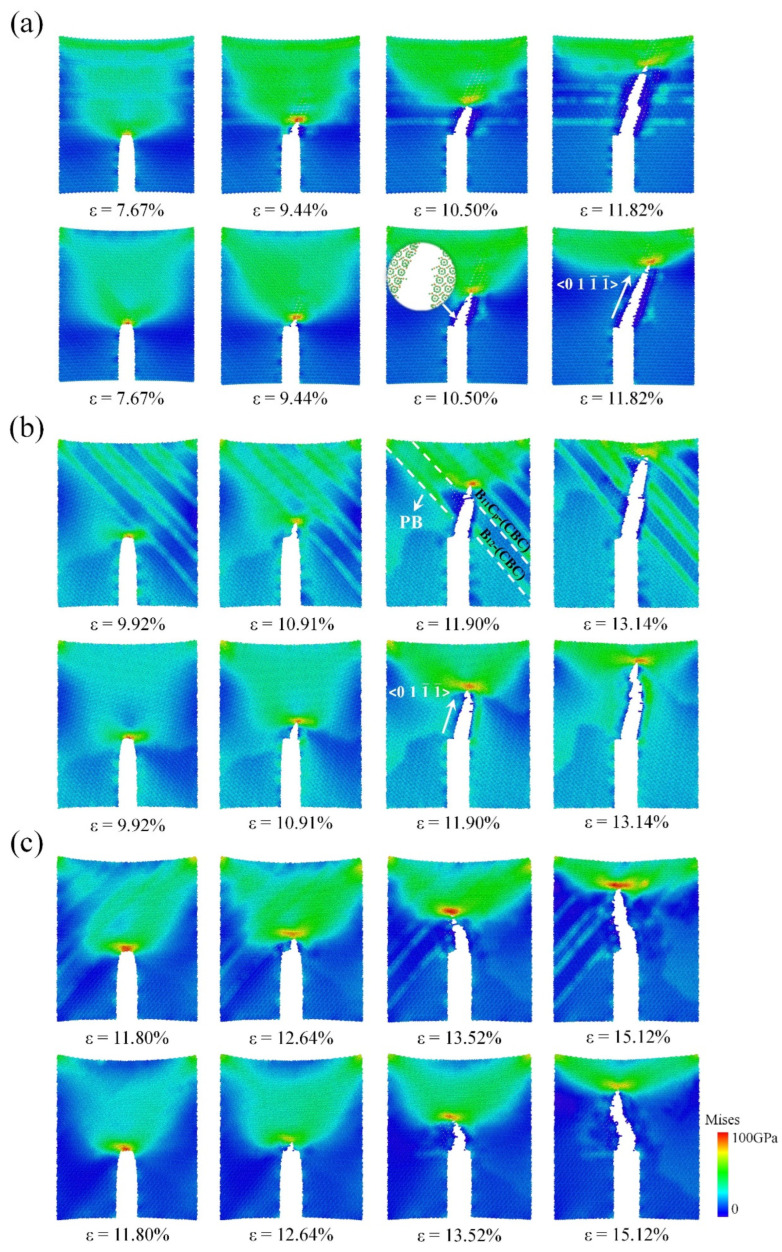
Changes in von Mises stress distribution with crack propagation for PB (first row) and SC (second row) B_4_C models at angles of (**a**) 90°, (**b**) 40°, and (**c**) 140°.

**Figure 8:**
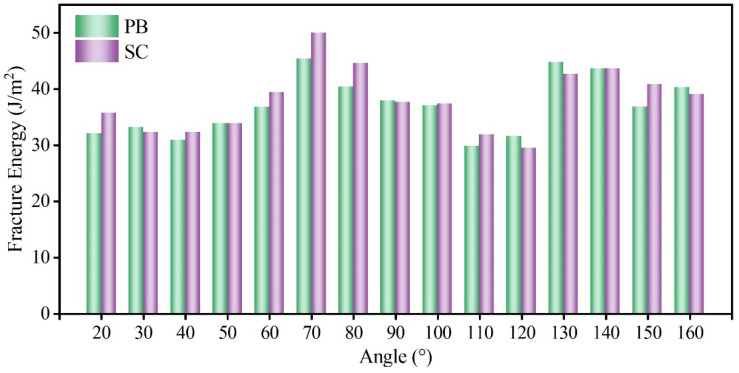
Variation in fracture energy of PB and SC B_4_C with included angle.

Figure 6 shows the stress–strain relationships of SC B_4_C and PB B_4_C at several typical included angles during the crack growth process. When the included angle is 90° or 140°, the stress–strain curves of SC B_4_C and PB B_4_C are basically consistent. This correspondence is further evidenced by the von Mises stress cloud diagrams in Figure 7, which show that the total lengths of crack propagation under the same fracture strain are nearly identical. The results indicate that when the prefabricated crack is perpendicular to the PB or the included angle is 140°, the presence of the PB has little effect on the fracture toughness of B_4_C. For the PB-40 model, although its fracture stress and fracture strain are almost consistent with those of the SC-40 model, its crack undergoes slight deflection during the propagation process. The local atomic structure shows that the crack continues to expand along the <$01\overset{-}{1}\overset{-}{1}$> crystal orientation after being deflected across the PB. Moreover, the crack deflects only when the crack propagates from the (B_12_)-CBC phase to the (B_11_C_p_)-CBC phase, as shown in Figure 7b. When the crack deflects, the stress decreases gently and the strain continues to increase, which manifests in the stress–strain curve as a step-like reduction in stress. As is well known, deflection of a crack increases the total length of crack propagation and absorbs more energy, thereby improving the toughness of the material. However, the calculated fracture energies of the SC-40 and PB-40 models are 30.83 J/m^2^ and 30.86 J/m^2^, respectively, which are almost identical. Therefore, the slight deflection caused by the crack crossing the PB has little effect on the fracture toughness of B_4_C. We calculate the fracture toughness of PB B_4_C as a function of the included angle, as shown in Figure 8. Similar to SC B_4_C, the fracture toughness of PB B_4_C is still greatly affected by the angle. The maximum and minimum values of the calculated fracture energy of PB B_4_C are 45.38 J/m^2^ and 29.83 J/m^2^, respectively. Note that the fracture energy of PB B_4_C is almost the same as that of SC B_4_C at the same included angle. Therefore, the existence of PBs has no toughening effect on B_4_C.

## 4. Conclusions

In the study reported herein, the fracture toughness and atomic-scale crack propagation process of NT B_4_C and PB B_4_C were investigated by MD simulations. The simulation results showed that the fracture toughness is greatly affected by the included angle whether it is SC, NT, or PB B_4_C. When the angle between the prefabricated crack and the TB is 40° or 100°–120°, the TB has a significant hindering effect on the expansion of cracks. The toughening effect of the TB reaches its maximum when the included angle is 120°, and its fracture toughness is 32.72% higher than that of SC B_4_C. Analysis of stress–strain relationships and von Mises stress cloud diagrams of crack propagation showed that the toughening effect of twins originates from the difference in fracture strength of SC B_4_C on both sides of the TB. The TB effectively obstructs the crack only when it transitions from a low-strength to a high-strength region. For PB B_4_C, although the crack slightly deflects when crossing the PB, the calculated fracture energy closely aligns with that of SC B_4_C, indicating that the PB has little effect on the toughness of B_4_C. Overall, the excellent toughening properties of nanotwins provide a novel strategy for producing B_4_C ceramics with high fracture toughness.

## Data Availability

The data that support the findings of this study are available from the corresponding author upon reasonable request.

## Supplementary Materials

The following supporting information can be downloaded at: https://www.mdpi.com/article/10.3390/nano14181493/s1, Figure S1: Stress-strain relationship of NT-90 model at different loading rates; Figure S2: (a) Atomic structures of asymmetric twin boundary, (b) Simulation models for fracture toughness of asymmetric twin, (c) Stress-strain relationship of NT-120 model and a-TB-120 model.
