# Supplementary material for: Toughening Mechanism in Nanotwinned Boron Carbide: A Molecular Dynamics Study
Source: Nanomaterials (Basel). 2024 Sep 14;14(18):1493. doi: 10.3390/nano14181493 (PMC11435336; doi:10.3390/nano14181493)
Supplement: Supplementary file 1 [file nanomaterials-14-01493-s001.zip › nanomaterials-3191025-supplementary.pdf]

Supplementary Materials

1. Effect of loading rate

We applied different loading rates of 1×10^{-5} Å/fs, 2.5×10^{-5} Å/fs, and 5×10^{-5} Å/fs to the NT-90 model to calculate the effect of loading rate on the stress-strain relationship. The calculated stress-strain relationship of NT-90 model with different loading rates is shown in Fig. S1.

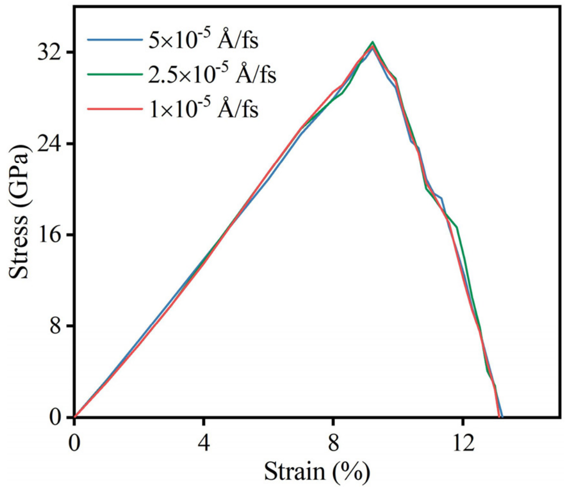

Fig. S1. Stress-strain relationship of NT-90 model at different loading rates

It can be seen from Figure S1 that the shear stress-strain curves of NT-90 model at different loading rates are almost consistent. Therefore, the loading rate has little influence on the research in this paper. It indicates that it is reliable to use loading strain rate of 5×10^{-5} Å/fs to calculate the stress-strain relationship of crack growth in the MD calculations of this study.

2. Calculation of fracture toughness of asymmetric twins

The special atomic arrangement of the boron carbide unit cell results in a special asymmetric twin (a-TB). The reason why the twins are asymmetric is that the polar boron carbide ($B_{11}C_P$-CBC) on the other side of the twin boundary becomes the

equatorial boron carbide ($B_{11}C_e$-CBC). Therefore, asymmetric twins can also be regarded as a special phase boundary composed of phase $B_{11}C_P$-CBC and phase $B_{11}C_e$-CBC. We established a fracture model of asymmetric twins on the same scale as symmetric twins and calculated the stress-strain curves of the fracture process of the asymmetric twin structure, as shown in Figure S2.

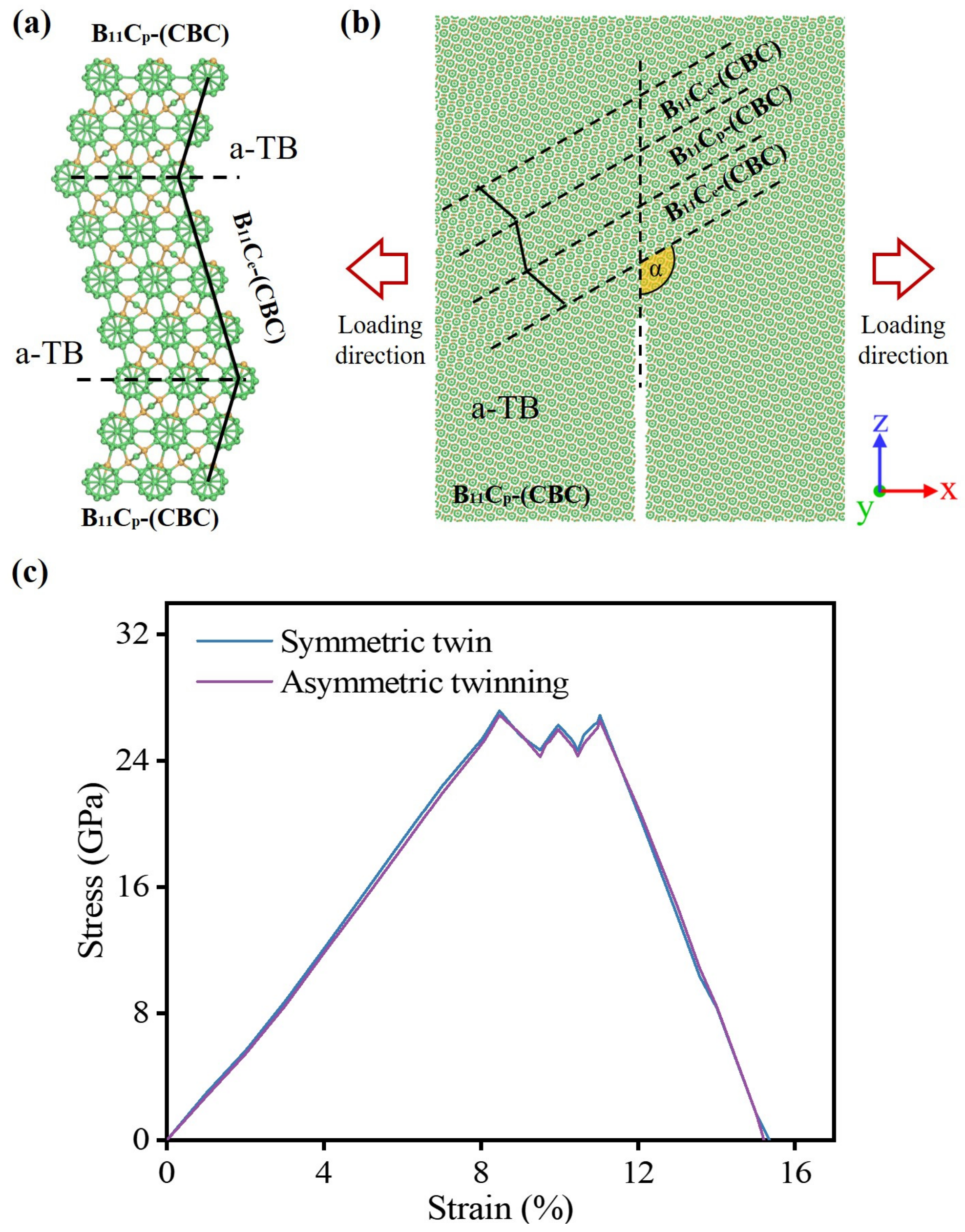

Fig. S2. (a) Atomic structures of asymmetric twin boundary, (b) Simulation models for fracture toughness of asymmetric twin, (c) Stress-strain relationship of NT-120 model and a-TB-120 model.

The MD simulation results show that the stress-strain curves of symmetric twins and asymmetric twins are basically consistent when the angle is 120°. Therefore, asymmetric twins also have the effect of hindering crack propagation and the toughening mechanism is the same as that of symmetric twins. The calculated fracture energies of symmetric twins and asymmetric twins are 39.07 J/m^2 and 38.92 J/m^2, respectively. Therefore, the toughening effect of asymmetric twins is basically the same as that of symmetric twins.
